# Comparison of the Multiple Platforms to Identify Various *Aeromonas* Species

**DOI:** 10.3389/fmicb.2020.625961

**Published:** 2021-01-18

**Authors:** Xiaoli Du, Mengyu Wang, Haijian Zhou, Zhenpeng Li, Jialiang Xu, Zhe Li, Biao Kan, Daoli Chen, Xiaoli Wang, Yujuan Jin, Yan Ren, Yanping Ma, Jiuyin Liu, Yang Luan, Zhigang Cui, Xin Lu

**Affiliations:** ^1^State Key Laboratory of Infectious Disease Prevention and Control, National Institute for Communicable Disease Control and Prevention, Chinese Center for Disease Control and Prevention, Beijing, China; ^2^School of Public Health, Nanchang University, Nanchang, Jiangxi, China; ^3^Jiangxi Provincial Key Laboratory of Preventive Medicine, Nanchang University, Nanchang, China; ^4^School of Light Industry, Beijing Technology and Business University, Beijing, China; ^5^Department of Microbiology Laboratory, Maanshan Center for Disease Control and Prevention of Anhui Province, Maanshan, China; ^6^Shijiazhuang Center for Disease Control and Prevention, Shijiazhuang, China; ^7^Longgang Center for Disease Control and Prevention, Shenzhen, China; ^8^LongHua District Center for Disease Control and Prevention, Shenzhen, China; ^9^Nanshan Center for Disease Control and Prevention, Shenzhen, China; ^10^Liaocheng Center for Disease Control and Prevention, Liaocheng, China; ^11^Xi'an Center for Disease Control and Prevention, Xi'an, China

**Keywords:** *Aeromonas*, whole-genome sequencing, mass spectrometry, multilocus phylogenetic analysis (MLPA), traditional biochemical testing, multiplex-PCR

## Abstract

We compared several identification methods for *Aeromonas* genus members, including traditional biochemical testing, multiplex-PCR amplification, mass spectrometry identification, whole-genome sequencing, multilocus phylogenetic analysis (MLPA), and *rpoD, gyrA*, and *rpoD*-*gyrA* gene sequencing. Isolates (*n* = 62) belonging to the *Aeromonas* genus, which were came from the bacterial bank in the laboratory, were used to assess the identification accuracy of the different methods. Whole-genome sequencing showed that the *Aeromonas* spp. isolates comprised *A. caviae* (*n* = 21), *A. veronii* (*n* = 18), *A. dhakensis* (*n* = 8), *A. hydrophila* (*n* = 7), *A. jandaei* (*n* = 5), *A. enteropelogenes* (*n* = 2), and *A. media* (*n* = 1). Using the whole-genome sequencing results as the standard, the consistency of the other methods was compared with them. The results were 46.77% (29/62) for biochemical identification, 83.87% (52/62) for mass spectrometric identification, 67.74% (42/62) for multiplex-PCR, 100% (62/62) for MLPA typing, 72.58% for *gyrA*, and 59.68% for *rpoD* and *gyrA*-*rpoD*. MLPA was the most consistent, followed by mass spectrometry. Therefore, in the public health laboratory, both MLPA and whole-genome sequencing methods can be used to identify various *Aeromonas* species. However, rapid and relatively accurate mass spectrometry is recommended for clinical lab.

## Introduction

*Aeromonas*, a Gram-negative, opportunistic pathogen bacterium, is prevalent in animals and the environment (Nolla-Salas et al., 2017). *Aeromonas* is often isolated from marine or aquatic organisms, and as an important fish pathogen, it causes septicemia and death in severe cases (Hossain et al., 2020). In 1963, *Aeromonas* was isolated from the blood of a girl with leukemia, which suggested its clinical significance (Bulger and Sherris, 1966). With increasing numbers of clinical cases of *Aeromonas*-related cases, this species is now considered to be a new gastrointestinal disease-causing pathogen in humans and other animals, and infections with it can become serious (Janda and Abbott, 2010). Diarrhea and food poisoning caused by *Aeromonas* have drawn increasing attention as foodborne illnesses (Pablos et al., 2010; Jamal et al., 2014). *Aeromonas* was previously classified as *Vibrio*, but phylogenetic studies have shown that it belongs to the *Aeromonas* genus. There are at least 18 *Aeromonas* species (Figueras et al., 2011), including *A. hydrophila, A. salmonicida, A. bestiarum, A. sobria, A. trota, A. caviae, A. popoffii, A. media, A. encheleia, A. veronii, A. aquariorum, A. eucrenophila, A. molluscorum, A. schubertii, A. simiae, A. jandaei, A. tecta*, and *A. bivalvium*. Three *A. hydrophila* subspecies exist among them. In 2010, four new species, *A. diversa* (Miñana-Galbis et al., 2010), *A. rivuli* (Figueras et al., 2011), *A. taiwanensis* (Alperi et al., 2010), and *A. sanarellii* (Alperi et al., 2010), were proposed. Altogether, 32 *Aeromonas* species have been identified to date (Martinez-Murcia et al., 2011), among which *A. caviae, A. hydrophila*, and *A. veronii* are closely associated with the clinical symptoms of diarrhea (Janda and Duffey, 1988; Parker and Shaw, 2011; Li et al., 2015). However, the specific *Aeromonas* types are relatively complex, and there is currently a lack of comprehensive and effective identification methods for them.

Traditional biochemical identification is a simple and low-cost method for preliminarily identifying *Aeromonas* members. However, identifying *Aeromonas* complex or new species requires supplementary experiments to be performed, so it is currently not possible to confidently distinguish *Aeromonas* species (Borrell et al., 1998; Martínez-Murcia et al., 2005; Janda and Abbott, 2010). Compared with traditional biochemical identification, mass spectrometry with its fast speed, simplicity, and high accuracy, is increasingly used to identify microorganisms (Bizzini et al., 2010; Benagli et al., 2012). With the development of biotechnology, multiplex-PCR is also commonly used for *Aeromonas* identification (Del Cerro et al., 2002; LaFrentz et al., 2019). Whole-genome sequencing technology has been widely used in various fields to accurately identify bacterial species by comparing the whole-genome sequences it generates; thus, this technique has become the reference method for bacterial species identification (Jamal et al., 2014; Hughes et al., 2016; Bartkova et al., 2017). MLPA typing is used to identify the characteristics of microbial isolates. This method assesses the degree of bacterial variation in a sample according to the differences existing among house-keeping gene sequences (Maiden et al., 1998; Martínez-Murcia et al., 2016). MLPA provides a strong species description framework for reliable, simple, and rapid identification of *Aeromonas* species (Navarro and inez-Murcia, 2018).

In this study, we compared the consistency of various techniques (i.e., biochemical detection, mass spectrometry identification, multiplex-PCR, MLPA, and *rpoD, gyrA, rpoD-gyrA* house-keeping gene amplification) with that of whole-genome sequencing at identifying *Aeromonas* species. The aim is to provide suggestion to choose the different method for the identification of *Aeromonas* according to different laboratory conditions.

## Materials and Methods

### Samples of *Aeromonas* spp.

Isolates belonging to the *Aeromonas* genus were came from the bacterial bank in the laboratory. Each sample was individually placed into alkaline peptone water broth (Beijing Land Bridge Co., Ltd., China) for 18–24 h at 37°C, and the mixture was inoculated onto an RS selective medium plate (Thermo Fisher Scientific, Massachusetts, USA) for 18–24 h at 37°C. Suspicious colonies on the RS Medium were selected and inoculated onto LB medium. Single colonies were selected and cultured for 18–24 h at 37°C (Soltan Dallal et al., 2016). The SYBR green fluorescence-based PCR method was used to rapidly screen for the presence of *Aeromonas* (Du et al., 2020).

### Biochemical Identification

Pure *Aeromonas* single colonies that developed within 18–24 h were picked using sterile absorbent cotton sticks dipped in a solution containing sterile 0.45% NaCl and with uniform grinding each was adjusted to 0.5 McNamara turbidity using the VITEK II (BioMerieux, Lyon, France) automatic biochemical identification card for Gram-negative bacteria on the identification apparatus. The quality control strain was *E. coli* (ATCC700323). The results were read according to the manufacturer's instructions.

### Multiplex PCR for *Aeromonas* Identification

Multiplex PCR was conducted in 50 μl volumes, with each reaction containing 25 μl of 2× Taq PCR MasterMix (TaKaRa, Dalian, China), 10 μ mol/L of upstream and downstream primers (Sangon, Shanghai, China), 2 μl of DNA template, with ddH_2_O used to make up the total volume (Persson et al., 2015).

### Mass Spectrometric Identification of *Aeromonas*

The matrix-assisted laser desorption/ionization time of flight mass spectrometry (MALDI-TOF MS) system used to type the 62 pure *Aeromonas* colonies was based on protein “fingerprints” (Lauková et al., 2018), and was performed using a Microflex MALDI-TOF MS mass spectrometer (Antobio, China). To this end, every single colony was mixed with matrix solution and completely dried, and the MALDI-TOF MS instructions were followed for testing. Results were evaluated using the Autof ms1000 (Antobio, China) identification database. The results were exported for local preservation and statistical analysis. An appraisal credibility score of > 95% was considered to be reliable in this study (Jamal et al., 2014).

### Genome-Wide Phylogenetic Analysis

The Wizard Genomic DNA Purification Kit (Promega, Madison, USA) was used to extract genomic DNA from the cultured strains. The Illumina HiSeq-^TM^2000 sequencing platform was used to conduct PE-150 double-terminal sequencing, and the size of the inserted fragments was 350 bp.

The published *Aeromonas* genome assembly sequence (32 *Aeromonas* species in total) was downloaded from the GenBank database (until July 25, 2019). Altogether, 364 *Aeromonas* genome sequences were included in the analysis. First, the 364 individually downloaded *Aeromonas* sequences were used to construct evolutionary trees, and some species were found to be on the same branch (Supplementary Figure 1). The *A. hydrophila* ATCC 7966 genome sequence (Accession: GCA_000014805-1) was used as the reference. Next, the sequences from our 62 strains and the 364 whole-genome sequences from GenBank were compared with the reference sequence, and the core genome and single-nucleotide polymorphism (SNP) mutation sites were identified using Mummer v3.23 software. Sites in the repeat region that were identified by Blastn v2.2.28 were removed. Based on the identified 103,037 SNPs, Fasttree v2.1.7 software was used to construct a maximum-likelihood tree, which was visualized using Figtree v1.4.3 software and the iTol website.

In this study, the average nucleotide identity (ANI) method was used to assess the sequence similarities among the 62 *Aeromonas* strains that we sequenced and the 364 whole-genome sequences from GenBank. Gegenees v.2.2.1 was then used to draw a heat map from this data, from which the consistency between the two methods was compared.

### MLPA Identification of *Aeromonas*

Blastn comparison software was used to identify the genomic location information for *gyrB, recA, dnaJ, gyrA, dnaX, atpD*, and *rpoD* house-keeping genes in the 426 strains, and the corresponding gene sequences from each strain were extracted using a perl script. We used mafft v7.123b software to perform a multi-sequence comparison on the sequences of the aforementioned seven housekeeping genes and the *gyrA* gene, *rpoD* gene, and *rpoD-gyrA* (Martinez-Murcia et al., 2011). The maximum-likelihood tree constructed by Fasttree v2.1.7 was visualized in Figtree (v1.4.3) software and the iTol website.

### Statistical Analysis

We used the Kappa index to analyze the consistency of two qualitative observations from the same subjects (Murphy-Zane and Pyle, 2018). The Kappa index does not only test the consistency of the two results, but also provides a measurement of the degree of consistency. The value range for the Kappa index is 0–1. It is generally believed that a Kappa value ≤ 0.4 shows a poorly consistent result, a value of 0.4 < Kappa ≤ 0.75 shows good consistency, and a Kappa value of ≥0.75 has the best consistency. Statistical significance was defined as *P* < 0.05. The Kappa index was used to compare the consistency of the *Aeromonas* identification methods with that of the whole-genome sequencing results.

## Results

### Identification of *Aeromonas*

In this study, the 62 isolates were assessed using the SYBR green fluorescence PCR method, the results of which confirmed that the 62 strains we isolated were *Aeromonas* spp.

### Species Identification by Whole-Genome Sequencing

**Figure 1:**
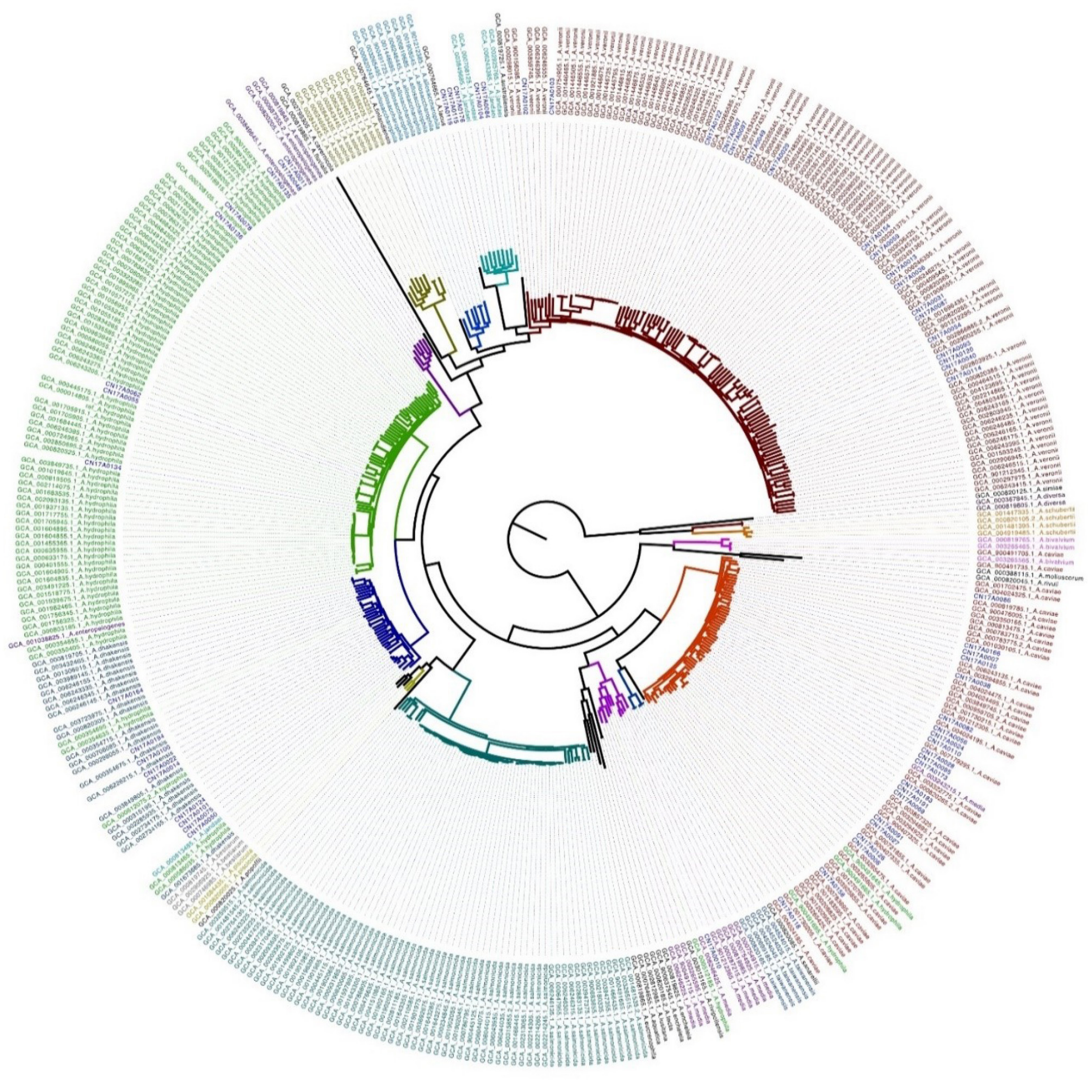
Maximum-likelihood tree based on genome-wide SNPs constructed for the 62 strains sequenced in this study versus 364 strains downloaded from Genbank.

**Figure 2:**
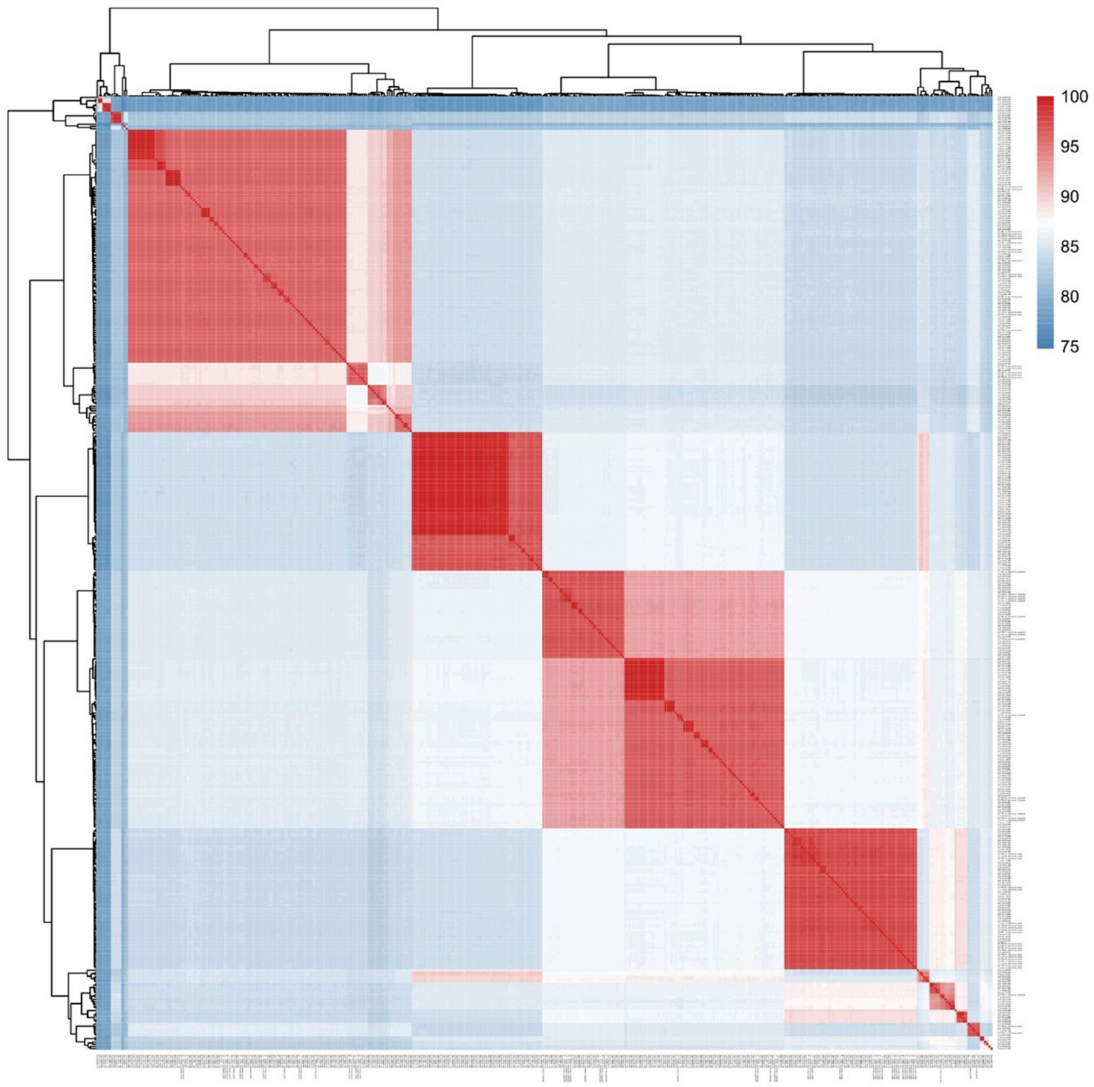
Phylogenomic analysis of the *Aeromonas* spp. examined in this study. The values generated by the Gegenees software shown in the heat map indicate the percentage similarity between the analyzed genomes. The colors vary from blue (low similarity) to red (high similarity).

A core genomic SNP tree was constructed on the 364 whole genomes we downloaded from GenBank, along with the genome sequences obtained in this study. According to the cluster generated by MLtree, the species of the 62 isolates from this study were defined as being on the same branch, in accordance with the known species of the 364 strains from GenBank. Finally, seven species in the *Aeromonas* genus were identified as *A. caviae, A. veronii, A. dhakensis, A. hydrophila, A. jandaei, A. enteropelogenes*, and *A. media* (Figure 1). The ANI method showed intraspecies nucleotide similarity rates of >97% (different strains within the same species) (Janda and Abbott, 2010). These results (Figure 2) are consistent with those of the SNP phylogenetic tree.

### Biochemical Identification

Compared with the whole-genome sequencing results, the overall accuracy was 46.77% (29/62) (Table 1). The Kappa index value, which at 0.378 is <0.4, showed poor consistency (Table 2). The best biochemical identification accuracy was for *A. hydrophila* and *A. caviae* (100% accuracy), with the worst accuracy being 0% for *A. dhakensis*. Moreover, *A. dhakensis* was identified as *A. caviae* 25% (2/8) or *A. hydrophila* 75% (6/8). The biochemical identification readily but mistakenly identified *A. jandaei, A. veronii*, and *A. enteropelogenes* as *A. sobria*. Three strains of *A. veronii* were identified as *A. hydrophila*, and one was not identifiable (Supplementary Table 1).

**Table 1:** Comparison of the identification accuracy of different methods for *Aeromonas* species.

**WGS result**	**Number**	**MLPA(%)**	**MS(%)**	**VITEK(%)**	**Multiplex-PCR(%)**	***rpoD*(%)**	***gyrA*(%)**	***gyrA and rpoD*(%)**
*A.dhakensis*	8	8(100)	0(0)	0(0)	0(0)	0(0)	0(0)	0(0)
*A.enteropelogenes*	2	2(100)	2(100)	0(0)	0(0)	2(100)	2(100)	2(100)
*A.jandaei*	5	5(100)	3(60)	0(0)	0(0)	2(40)	5(100)	2(40)
*A.hydrophila*	7	7(100)	7(100)	7(100)	7(100)	6(85.7)	5(71.4)	6(85.7)
*A.caviae*	21	21(100)	18(85.7)	21(100)	19(90.5)	14(66.7)	18(85.7)	14(66.7)
*A.veronii*	18	18(100)	18(100)	2(11.1)	16(88.9)	9(50)	17(94.4)	9(50)
*A.media*	1	1(100)	1(100)	0(0)	1(100)	1(100)	1(100)	1(100)
*No. of isolates identified (%)*	–	62(100)	52(83.9)	29(46.8)	42(67.7)	37(59.7)	45(72.6)	37(59.7)

**Table 2:** Kappa values of the various methods vs. the whole-genome sequencing results.

	**MLPA**	**MS**	***gyrA***	**Multi-PCR**	***rpoD***	***gyrA-rpoD***	**VITEK**
Kappa	1	0.73	0.689	0.597	0.469	0.469	0.378
Consistency	Best	Good	Good	Good	Good	Good	Poor

### Multiplex-PCR

Compared with the whole-genome sequencing results, the multiplex-PCR accuracy was 67.74% (42/62) (Table 1). At 0.597, the Kappa index is <0.75 but shows good consistency (Table 2). Among them, the identification of *A. media* and *A. hydrophila* was 100% accurate. Both *A. enteropelogenes* and *A. jandaei* were identified as *A. veronii* by multiplex PCR, but *A. dhakensis* was not identified by this method (Supplementary Table 1).

### Mass Spectrometry

Taking the whole-genome sequencing results as the reference standard, the accuracy of mass spectrometry testing was 83.87% (52/62) (Table 1), and 45 strains scored above 95. At 0.73, the Kappa index is <0.75 but shows good consistency (Table 2). The accuracy of *A. veronii* identification was 100% (18/18, Supplementary Table 1). Mass spectrometry typed *A. caviae* as *A. hydrophila*, and could not identify *A. dhakensis* at all (Supplementary Table 1).

### *rpoD, gyrA*, and *rpoD-gyrA* Sequence Typing

In this study, the results from *rpoD* and *rpoD-gyrA* identification were consistent. Compared with the whole-genome sequencing results, the accuracy of *rpoD* and *gyrA* gene identification was 59.7% (37/62) and 72.6% (45/62), respectively (Table 1), and the Kappa index values were 0.469 and 0.689, respectively, which although below 0.75, had good consistency (Table 2). The consistency rate for the *gyrA* gene method was 100% (5/5) for the identification of *A. jandaei* (Table 1). Both *rpoD-gyrA* and *rpoD* showed 100% consistency at identifying *A. enteropelogenes* (Table 1), but neither gene distinguished *A. jandaei* from *A. caviae* (Supplementary Table 1).

### MLPA Typing

As shown in Table 1, based on the MLPA phylogenetic analysis of the seven house-keeping genes, seven species in the *Aeromonas* genus were identified. The results of the MLPA typing were 100% consistent with the genome-wide identification results. The Kappa index showed this method to have the best consistency of all the tested methods (Table 2).

## Discussion

Heterogeneity in the phenotypes and genotypes of *Aeromonas* makes species identification in this genus very complicated (Janda and Abbott, 2010). The emergence of various identification methods has helped with the identification process. Nevertheless, factors relating to the identification method itself and interference from various factors in the identification process have created discrepancies in accuracy among the various methods. In this study, the consistency rates among the multiple methods we used for identifying *Aeromonas* species were compared with the results from genome-wide identification, and the advantages and disadvantages of these methods in their ability to accurately identify *Aeromonas* species were evaluated.

The results of the evolutionary tree prepared from the whole-genome sequences showed that our 62 *Aeromonas* isolates fell into seven species: *A. caviae* 33.9% (21/62), *A. veronii* 29.0% (18/62), *A. hydrophila* 12.9% (8/62), *A. dhakensis* 11.3% (7/62), *A. jandaei* 8.1% (5/62), *A. enteropelogenes* 3.2% (2/62), and *A. media* 1.6% (1/62). Previous studies have shown MLPA typing to be consistent with the results from whole-genome sequencing (Martinez-Murcia et al., 2011). In the present study, MLPA typing showed the highest degree of consistency with the whole-genome sequencing results when compared with the other methods. *rpoD* and *gyrA* genes were used separately to type *Aeromonas*, and the result for *gyrA* was more consistent with the whole-genome sequencing results than that of *rpoD* (Table 1). Li et al. (Xinyue et al., 2016) speculated that this type of result may be related to the fact that *rpoD* is not good at distinguishing *A. allosaccharophila* from *A. jandaei*. Our study found that the results from *rpoD*-*gyrA* accorded with those from *rpoD* alone. Furthermore, the results from *gyrA, rpoD*, and *rpoD*-*gyrA* showed that they could not distinguish *A. caviae* from *A. veronii*, a result consistent with that from Persson et al. (2015) and Beaz-Hidalgo et al. (2010), but the identification of *A. enteropelogenes* was 100% (2/2). Although *rpoD* could not distinguish *A. allosaccharophila* from *A. jandaei, rpoD* had a higher consistency rate than *gyrA* at identifying *A. hydrophila*. With *A. caviae*, the biochemical identification showed 100% agreement with that for the whole genome. Except for *A. hydrophila* and *A. caviae*, where the accuracy of the biochemical identification was 100% for both, all other *Aeromonas* species were 0%, a finding consistent with the conclusion of Zhou (Yanyan et al., 2019).

Mass spectrometry identification is based on the unique map of protein peaks available in a commercial database (Benagli et al., 2012). The consistency between mass spectrometry identification and that of whole-genome sequencing was 83.9%, a result that may be related to the updated commercial database. Because *A. dhakensis* is a newly identified species, the database was not updated at the time we conducted this study, which led to a failure of identification. Despite multiplex PCR failing to amplify all of the target genes, its concordance rate with the whole-genome sequencing results was between that of biochemical identification and mass spectrometric identification. Multiplex-PCR technology misidentified *A. enteropelogenes* and *A. jandaei* as *A. veronii*. It has been reported that biochemical identification, mass spectrometry, and multiplex-PCR methods can accurately identify *A. hydrophila* (Bulger and Sherris, 1966; Wang et al., 2008; Elbehiry et al., 2019). In our study, the identification accuracy of *A. hydrophila* by the biochemical identification and multiplex-PCR methods was also 100%. The mass spectrometry identification method readily misidentified *A. caviae* as *A. hydrophila* (error rate, 14.3%), a finding consistent with the conclusion from a published study (Yanyan et al., 2019).

The whole-genome sequencing method used herein redefined *A. dhakensis* (obtained from human wounds), which was previously wrongly classified as *A. hydrophila* (Sinclair et al., 2016). When the virulence of *A. hydrophila* was compared with that of *A. dhakensis*, it was found that *A. dhakensis* was more virulent than *A. hydrophila* (Chen et al., 2013). Use of whole-genome sequencing technology should counteract species identification errors over time, thereby helping to make clinical diagnosis more accurate. Except for MLPA typing, the other identification methods misidentified *A. dhakensis* as *A. hydrophila* or *A. aquariorum* when compared with the whole-genome sequencing results, probably because *A. dhakensis* was originally considered a closely related subtype of *A. hydrophila* (Figueras et al., 2011). Furthermore, in one study, the identification rate for *A. dhakensis* based on its unique protein peak was 96.7% (Chen et al., 2014). In the present study, the mass spectrometer could not distinguish *A. dhakensis* from *A. hydrophila*, suggesting that the optimization of the protein peak diagram in the commercial database is conducive to the identification of *Aeromonas* species by mass spectrometry. A domestic study showed that the accuracy rate for mass spectrometry for *A. enteropelogenes* was 100 and 96.7% for *A. media* (Yanyan et al., 2019). In the present study, all the identification methods were able to identify *A. media* 100% (1/1), except for the biochemical identification method (Table 1). However, this result will need further confirmation because of the small sample size in this study.

When compared with the whole-genome sequencing results, the accuracy of MLPA typing was the highest of all the tested methods, attaining 100% for all 62 of the isolates. Previous studies have shown that the MLPA method achieves results that are consistent with those from whole-genome sequencing, and that the MLPA method can be widely used to screen and species identify isolated bacteria (Navarro and inez-Murcia, 2018). The consistency rate between mass spectrometry identification and whole-genome sequencing identification was 83.87%, somewhat lower than MLPA typing. We also found that the traditional biochemical identification method for *A. hydrophila* and *A. caviae* is better than that of mass spectrometry. Because of the difficulty in identifying other *Aeromonas* species, we suggest that biochemical identification is used for identifying *Aeromonas* genus members. While multiplex-PCR technology has some ability to identify common *Aeromonas* species such as *A. caviae* and *A. veronii*, the *rpoD* or *gyrA* method can be used for uncommon species such as *A. enteropelogenes, A. jandaei*, and *A. media*. That one previous study has also reported on a poor consistency of identification from multiple methods (Ørmen et al., 2005) indicates that a variety of identification methods should be combined for *Aeromonas* species identification. Due to the limitation of sample size in this study, a larger sample size is needed for confirmation.

With differences in accuracy between various methods clearly existing, the whole-genome sequencing method provides a unified standard with which to compare the various methods. Currently, biochemical identification is mainly used in clinical practice to identify isolated bacteria, but it is a time-consuming and laborious method, and the identification results are not accurate enough, which leads to diagnostic misjudgment (Jamal et al., 2014). If the commercial database of mass spectrometry is updated on time, the consistency with whole-genome sequencing results will be improved. What's more, the cost of mass spectrometric identification is reasonable and its operation straightforward, it can be used in the clinical lab to preliminarily identify *Aeromonas* members. As sequencing technology became more and more convenient in the public health labortory, both MLPA and whole-genome sequencing methods can be used to identify various *Aeromonas* species. Therefore, choosing an appropriate method for identifying *Aeromonas* species needs to be situation specific.

## Data Availability Statement

The datasets presented in this study can be found in online repositories. The names of the repository/repositories and accession number(s) can be found below: NCBI GenBank, accession no: PRJNA685342.

## Author Contributions

XD and MW: wrote the editorial. HZ, Zhenl, JX, ZheL, and BK: provided technical assistance. DC, XW YJ, YR, YM, JL, and YL: performed data analysis and prepared the resources. ZC and XL: edited the editorial. All the authors read and approved the editorial.

## Conflict of Interest

The authors declare that the research was conducted in the absence of any commercial or financial relationships that could be construed as a potential conflict of interest.

## References

[B1] Alperi A. Martínez-Murcia A. J. Ko W. C. Monera A. Saavedra M. J. Figueras M. J. (2010). *Aeromonas taiwanensis* sp. nov. and *Aeromonas sanarellii* sp. nov., clinical species from Taiwan. Int. J. Syst. Evol. Microbiol. 60, 2048–2055. 10.1099/ijs.0.014621-0 19819994

[B2] Bartkova S. Leekitcharoenphon P. Aarestrup F. M. Dalsgaard I. (2017). Epidemiology of Danish *Aeromonas salmonicida* subsp. salmonicida in fish farms using whole genome sequencing. Front. Microbiol. 8:2411. 10.3389/fmicb.2017.02411 29259599 PMC5723325

[B3] Beaz-Hidalgo R. Alperi A. Buján N. Romalde J. L. Figueras M. J. (2010). Comparison of phenotypical and genetic identification of *Aeromonas* strains isolated from diseased fish. Syst. Appl. Microbiol. 33, 149–153. 10.1016/j.syapm.2010.02.002 20227844

[B4] Benagli C. Demarta A. Caminada A. Ziegler D. Petrini O. Tonolla M. (2012). A rapid MALDI-TOF MS identification database at genospecies level for clinical and environmental *Aeromonas* strains. PLoS One 7:e48441. 10.1371/journal.pone.0048441 23119019 PMC3485216

[B5] Bizzini A. Durussel C. Bille J. Greub G. Prod'hom G. (2010). Performance of matrix-assisted laser desorption ionization-time of flight mass spectrometry for identification of bacterial strains routinely isolated in a clinical microbiology laboratory. J. Clin. Microbiol. 48, 1549–1554. 10.1128/JCM.01794-09 20220166 PMC2863943

[B6] Borrell N. Figueras M. J. Guarro J. (1998). Phenotypic identification of *Aeromonas* genomospecies from clinical and environmental sources. Can. J. Microbiol. 44, 103–108. 10.1139/cjm-44-2-103 9575026

[B7] Bulger R. J. Sherris J. C. (1966). The clinical significance of *Aeromonas hydrophila*. Report of two cases. Arch. Intern. Med. 118, 562–564. 10.1001/archinte.118.6.562 5925443

[B8] Chen P.-L. Wu C.-J. Chen C.-S. Tsai P.-J. Tang H.-J. Ko W.-C. (2013). A comparative study of clinical *Aeromonas dhakensis* and *Aeromonas hydrophila* isolates in southern Taiwan: *A*. dhakensis is more predominant and virulent. Clin. Microbiol. Infect. 20, 428–434. 10.1111/1469-0691.12456 24237662

[B9] Chen P. L. Lee T. F. Wu C. J. Teng S. H. Teng L. J. Ko W. C.. (2014). Matrix-assisted laser desorption ionization-time of flight mass spectrometry can accurately differentiate *Aeromonas dhakensis* from hydrophila A, A. caviae, veronii A. J. Clin. Microbiol. 52, 2625–2628. 10.1128/JCM.01025-14 24759711 PMC4097716

[B10] Del Cerro A. Marquez I. Guijarro J. A. (2002). Simultaneous detection of *Aeromonas salmonicida*, Flavobacterium psychrophilum, *Yersinia ruckeri*. Three major fish pathogens, by multiplex PCR. Appl. Environ. Microbiol. 68, 5177–5180. 10.1128/AEM.68.10.5177-5180.2002 12324372 PMC126410

[B11] Du X. Liu J. Hong Y. Wang L. Jiang L. Chen J. (2020). Establishment and evaluation of SYBR green fluorescent PCR for detection of *Aeromonas*. Dis. Surveill. 35, 425–429. 10.3784/j.issn.1003-9961.2020.05.013

[B12] Elbehiry A. Marzouk E. Abdeen E. Al-Dubaib M. Alsayeqh A. Ibrahem M.. (2019). Proteomic characterization and discrimination of *Aeromonas* species recovered from meat and water samples with a spotlight on the antimicrobial resistance of *Aeromonas hydrophila*. Microbiologyopen. 8:e782. 10.1002/mbo3.782 30614207 PMC6854848

[B13] Figueras M. J. Alperi A. Beaz-Hidalgo R. Stackebrandt E. Brambilla E. Monera A.. (2011). *Aeromonas rivuli* sp. nov., isolated from the upstream region of a karst water rivulet. Int. J. Syst. Evol. Microbiol. 61, 242–248. 10.1099/ijs.0.016139-0 20207806

[B14] Hossain S. De Silva B. C. J. Dahanayake P. S. De Zoysa M. Heo G. J. (2020). Phylogenetic characteristics, virulence properties and antibiogram profile of motile *Aeromonas* spp. isolated from ornamental guppy (*Poecilia reticulata*). Arch. Microbiol. 202, 501–509. 10.1007/s00203-019-01762-5 31707424

[B15] Hughes H. Y. Conlan S. P. Lau A. F. Dekker J. P. Michelin A. V. Youn J. H.. (2016). Detection and whole-genome sequencing of carbapenemase-producing *Aeromonas hydrophila* isolates from routine perirectal surveillance culture. J. Clin. Microbiol. 54, 1167–1170. 10.1128/JCM.03229-15 26888898 PMC4809936

[B16] Jamal W. Albert M. J. Rotimi V. O. (2014). Real-time comparative evaluation of bioMerieux VITEK MS versus Bruker Microflex MS, two matrix-assisted laser desorption-ionization time-of-flight mass spectrometry systems, for identification of clinically significant bacteria. BMC Microbiol. 14:289. 10.1186/s12866-014-0289-0 25433488 PMC4290442

[B17] Janda J. M. Abbott S. L. (2010). The genus *Aeromonas*: taxonomy, pathogenicity, and infection. Clin. Microbiol. Rev. 23, 35–73. 10.1128/CMR.00039-09 20065325 PMC2806660

[B18] Janda J. M. Duffey P. S. (1988). Mesophilic aeromonads in human disease: current taxonomy, laboratory identification, and infectious disease spectrum. Rev. Infect. Dis. 10, 980–997. 10.1093/clinids/10.5.980 3055195

[B19] LaFrentz B. R. Garcia J. C. Shelley J. P. (2019). Multiplex PCR for genotyping *Flavobacterium columnare*. J. Fish Dis. 42, 1531–1542. 10.1111/jfd.13068 31469439

[B20] Lauková A. Kubašová I. Kandričáková A. Strompfová V. Žitnan R. Simonová M. P. (2018). Relation to enterocins of variable *Aeromonas* species isolated from trouts of Slovakian aquatic sources and detected by MALDI-TOF mass spectrometry. Folia Microbiol. (Praha). 63, 749–755. 10.1007/s12223-018-0616-1 29808450

[B21] Li F. Wang W. Zhu Z. Chen A. Du P. Wang R.. (2015). Distribution, virulence-associated genes and antimicrobial resistance of *Aeromonas* isolates from diarrheal patients and water, China. J. Infect. 70, 600–608. 10.1016/j.jinf.2014.11.004 25447712

[B22] Maiden M. C. J. Bygraves J. A. Feil E. (1998). Multilocus sequence typing: a portable approach to the identification of clones within populations of pathogenic microorganisms. Proc. Natl. Acad. Sci. U. S. A. 95, 3140–3145. 10.1073/pnas.95.6.3140 9501229 PMC19708

[B23] Martínez-Murcia A. Beaz-Hidalgo R. Navarro A. Carvalho M. J. Aravena-Román M. Correia A.. (2016). *Aeromonas lusitana* sp. nov., isolated from untreated water and vegetables. Curr. Microbiol. 72, 795–803. 10.1007/s00284-016-0997-9 26868258

[B24] Martinez-Murcia A. J. Monera A. Saavedra M. J. Oncina R. Lopez-Alvarez M. Lara E.. (2011). Multilocus phylogenetic analysis of the genus *Aeromonas*. Syst. Appl. Microbiol. 34, 189–199. 10.1016/j.syapm.2010.11.014 21353754

[B25] Martínez-Murcia A. J. Soler L. Saavedra M. J. Chacón M. R. Guarro J. Stackebrandt E.. (2005). Phenotypic, genotypic, and phylogenetic discrepancies to differentiate *Aeromonas salmonicida* from *Aeromonas bestiarum*. Int. Microbiol. 8, 259–269. 16562378

[B26] Miñana-Galbis D. Farfán M. Gaspar Lorén J. Carmen Fust,é M. (2010). Proposal to assign *Aeromonas diversa* sp. nov. as a novel species designation for *Aeromonas* group 501. Syst. Appl. Microbiol. 33, 15–19. 10.1016/j.syapm.2009.11.002 20005654

[B27] Murphy-Zane M. S. Pyle L. (2018). Reliability of the anterior humeral line index compared with the gartland classification for posteriorly hinged supracondylar humerus fractures. Orthopedics. 41, e502–e505. 10.3928/01477447-20180424-06 29708571

[B28] Navarro A. inez-Murcia A. M. (2018). Phylogenetic analyses of the genus *Aeromonas* based on housekeeping gene sequencing and its influence on systematics. J. Appl. Microbiol. 125, 622–631. 10.1111/jam.13887 29676027

[B29] Nolla-Salas J. Codina-Calero J. Valles-Angulo S. Sitges-Serra A. Zapatero-Ferrandiz A. Climent M. C.. (2017). Clinical significance and outcome of *Aeromonas* spp. infections among 204 adult patients. Eur. J. Clin. Microbiol. Infect. Dis. 36, 1393–1403. 10.1007/s10096-017-2945-4 28258303 PMC7102105

[B30] Ørmen Ø. Granum PE Lassen J. Figueras M. J. (2005). Lack of agreement between biochemical and genetic identification of *Aeromonas* spp. Food Saf. Infect. 113, 203–207. 10.1111/j.1600-0463.2005.apm1130308.x 15799764

[B31] Pablos M. Remacha M. A. Rodriguez-Calleja J. M. Santos J. A. Otero A. Garcia-Lopez M. L. (2010). Identity, virulence genes, and clonal relatedness of *Aeromonas* isolates from patients with diarrhea and drinking water. Eur. J. Clin. Microbiol. Infect. Dis. 29, 1163–1172. 10.1007/s10096-010-0982-3 20549532

[B32] Parker J. L. Shaw J. G. (2011). *Aeromonas* spp. clinical microbiology and disease. J. Infect. 62, 109–118. 10.1016/j.jinf.2010.12.003 21163298

[B33] Persson S. Al-Shuweli S. Yapici S. Jensen J. N. Olsen K. E. (2015). Identification of clinical *Aeromonas* species by rpoB and gyrB sequencing and development of a multiplex PCR method for detection of hydrophila A, caviae A, A. veronii, media A. J. Clin. Microbiol. 53, 653–656. 10.1128/JCM.01963-14 25411168 PMC4298543

[B34] Sinclair H. A. Heney C. Sidjabat H. E. George N. M. Bergh H. Anuj S. N.. (2016). Genotypic and phenotypic identification of *Aeromonas* species and CphA-mediated carbapenem resistance in Queensland, Australia. Diagn. Microbiol. Infect. Dis. 85, 98–101. 10.1016/j.diagmicrobio.2016.02.005 26971634

[B35] Soltan Dallal M. M. Mazaheri Nezhad Fard R. Kavan Talkhabi M. Aghaiyan L. Salehipour Z. (2016). Prevalence, virulence and antimicrobial resistance patterns of *Aeromonas* spp. isolated from children with diarrhea. Germs. 6, 91–96. 10.11599/germs.2016.1094 27622161 PMC5018390

[B36] Wang Y. Tang C. Yu X. Wang Y. Yue H. (2008). [Detecting pathogenic *Aeromonas hydrophila* in fish by triplex PCR]. Wei Sheng Wu Xue Bao. 48, 947–951. 18837375

[B37] Xinyue L. Fengjuan L. Pengcheng D. Ruibai W. Duochun W. (2016). Comparative analysis on identification of *Aeromonas* by rpoD and gyrA genes. Dis. Surveill. 31, 200–204. 10.3784/j.issn.1003-9961.2016.03.006

[B38] Yanyan Z. Zheng N. Donghui Y. (2019). Three methods to identify the effect of clinical isolation strain of *Aeromonas*. Dis. Surveill. 34, 70–75. 10.3784/j.issn.1003-9961.2019.01.017

