# Supplementary material for: Comparison of the Multiple Platforms to Identify Various Aeromonas Species
Source: Front Microbiol. 2021 Jan 18;11:625961. doi: 10.3389/fmicb.2020.625961 (PMC7848130; doi:10.3389/fmicb.2020.625961)
Supplement: Supplementary file 1 [file Data_Sheet_1.PDF]

SUPPLEMENTARY TABLE 1 Comparison of identification results of each method

Key	WGS	MLPA	MS	VITEK	Multi-PCR Species	*rpoD*	*gyrA*	*gyrA and rpoD*
CN17A0006	*A.caviae*	*A.caviae*	*A.caviae*	*A.caviae*	*A.caviae*	*A.caviae*	*A.caviae*	*A.caviae*
CN17A0007	*A.caviae*	*A.caviae*	*A.caviae*	*A.caviae*	*A.caviae*	*A.caviae*	*A.caviae*	*A.caviae*
CN17A0010	*A.media*	*A.media*	*A.media*	*A.caviae*	*A.media*	*A.media*	*A.media*	*A.media*
CN17A0011	*A.enteropelogenes*	*A.enteropelogenes*	*A.enteropelogenes*	*A.sobria*	*A.veronii*	*A.enteropelogenes*	*A.enteropelogenes*	*A.enteropelogenes*
CN17A0013	*A.veronii*	*A.veronii*	*A.veronii*	*A.sobria*	*A.veronii*	*A.veronii*	*A.veronii*	*A.veronii*
CN17A0014	*A.hydrophila*	*A.hydrophila*	*A.hydrophila*	*A.hydrophila*	*A.hydrophila*	*A.aquariorum*	*A.aquariorum*	*A.aquariorum*
CN17A0022	*A.hydrophila*	*A.hydrophila*	*A.hydrophila*	*A.hydrophila*	*A.hydrophila*	*A.aquariorum*	*A.hydrophila*	*A.aquariorum*
CN17A0024	*A.caviae*	*A.caviae*	*A.caviae*	*A.caviae*	*A.caviae*	*A.caviae*	*A.media*	*A.caviae*
CN17A0027	*A.caviae*	*A.caviae*	*A.caviae*	*A.caviae*	*A.caviae*	*A.caviae*	*A.caviae*	*A.caviae*
CN17A0028	*A.caviae*	*A.caviae*	*A.caviae*	*A.caviae*	*A.caviae*	*A.caviae*	*A.caviae*	*A.caviae*
CN17A0029	*A.veronii*	*A.veronii*	*A.veronii*	*A.veronii*	*A.veronii*	*A.veronii*	*A.veronii*	*A.veronii*
CN17A0031	*A.veronii*	*A.veronii*	*A.veronii*	*A.sobria*	*A.veronii*	*A. allosaccharophila*	*A.veronii*	*A. allosaccharophila*
CN17A0036	*A.veronii*	*A.veronii*	*A.veronii*	*A.hydrophila*	*A.veronii*	*A.veronii*	*A.veronii*	*A.veronii*
CN17A0038	*A.caviae*	*A.caviae*	*A.caviae*	*A.caviae*	*A.caviae*	*A.caviae*	*A.caviae*	*A.caviae*
CN17A0040	*A.veronii*	*A.veronii*	*A.veronii*	*A.sobria*	*A.veronii*	*A.veronii*	*A.veronii*	*A.veronii*
CN17A0048	*A.enteropelogenes*	*A.enteropelogenes*	*A.enteropelogenes*	*A.sobria*	*A.veronii*	*A.enteropelogenes*	*A.veronii*	*A.enteropelogenes*
CN17A0049	*A.veronii*	*A.veronii*	*A.veronii*	*A.hydrophila*	*unknown*	*A.veronii*	*A.veronii*	*A.veronii*
CN17A0050	*A.dhakensis*	*A.dhakens*	*A.hydrophila*	*A.hydroph*	*A.hydroph*	*A.aquario*	*A.aquario*	*A.aquarior*

		is		*ila*	*ila*	*rum*	*rum*	*um*
CN17A0054	*A.veronii*	*A.veronii*	*A.veronii*	*A.sobria*	*A.veronii*	*A. allosaccharophila*	*A.veronii*	*A. allosaccharophila*
CN17A0055	*A.hydrophila*	*A.hydrophila*	*A.hydrophila*	*A.hydrophila*	*A.hydrophila*	*A.hydrophila*	*A.veronii*	*A.hydrophila*
CN17A0056	*A.caviae*	*A.caviae*	*A.caviae*	*A.caviae*	*A.caviae*	*A.caviae*	*A.caviae*	*A.caviae*
CN17A0059	*A.veronii*	*A.veronii*	*A.veronii*	*A.sobria*	*A.veronii*	*A. allosaccharophila*	*A. allosaccharophila*	*A. allosaccharophila*
CN17A0062	*A.hydrophila*	*A.hydrophila*	*A.hydrophila*	*A.hydrophila*	*A.hydrophila*	*A.hydrophila*	*A.veronii*	*A.hydrophila*
CN17A0067	*A.veronii*	*A.veronii*	*A.veronii*	*unknown*	*A.veronii*	*A.veronii*	*A.veronii*	*A.veronii*
CN17A0069	*A.caviae*	*A.caviae*	*A.caviae*	*A.caviae*	*A.caviae*	*A.veronii*	*A.caviae*	*A.veronii*
CN17A0075	*A.dhakensis*	*A.dhakensis*	*A.hydrophila*	*A.caviae*	*A.hydrophila*	*A.aquariorum*	*A.veronii*	*A.aquariorum*
CN17A0078	*A.hydrophila*	*A.hydrophila*	*A.hydrophila*	*A.hydrophila*	*A.hydrophila*	*A.hydrophila*	*A.hydrophila*	*A.hydrophila*
CN17A0082	*A.caviae*	*A.caviae*	*A.caviae*	*A.caviae*	*A.caviae*	*A.jandaei*	*A.caviae*	*A.jandaei*
CN17A0084	*A.jandaei*	*A.jandaei*	*A.jandaei*	*A.sobria*	*A.veronii*	*A.caviae*	*A.jandaei*	*A.jandaei*
CN17A0086	*A.caviae*	*A.caviae*	*A.caviae*	*A.caviae*	*A.caviae*	*A. allosaccharophila*	*A.caviae*	*A.jandaei*
CN17A0087	*A.veronii*	*A.veronii*	*A.veronii*	*A.sobria*	*A.veronii*	*A.caviae*	*A.veronii*	*A.jandaei*
CN17A0091	*A.caviae*	*A.caviae*	*A.caviae*	*A.caviae*	*A.caviae*	*A. allosaccharophila*	*A.caviae*	*A.jandaei*
CN17A0093	*A.veronii*	*A.veronii*	*A.veronii*	*A.sobria*	*A.veronii*	*A.caviae*	*A.veronii*	*A.caviae*
CN17A0095	*A.caviae*	*A.caviae*	*A.caviae*	*A.caviae*	*A.caviae*	*A.veronii*	*A.caviae*	*A.veronii*

CN17A0097	*A.veronii*	*A.veronii*	*A.veronii*	*A.sobria*	*unknown*	*A.aquariorum*	*A.veronii*	*A.aquariorum*
CN17A0100	*A.dhakensis*	*A.dhakensis*	*A.hydrophila*	*A.hydrophila*	*A.hydrophila*	*A. allosaccharophila*	*A.aquariorum*	*unknown*
CN17A0101	*A.dhakensis*	*A.dhakensis*	*A.hydrophila*	*A.hydrophila*	*A.hydrophila*	*A. allosaccharophila*	*A.aquariorum*	*unknown*
CN17A0102	*A.veronii*	*A.veronii*	*A.veronii*	*A.sobria*	*A.veronii*	*A.jandaei*	*A.veronii*	*A.jandaei*
CN17A0103	*A.veronii*	*A.veronii*	*A.veronii*	*A.sobria*	*A.veronii*	*A.jandaei*	*A.veronii*	*A.jandaei*
CN17A0104	*A.jandaei*	*A.jandaei*	*A.hydrophila*	*A.sobria*	*A.veronii*	*A.jandaei*	*A.jandaei*	*A.jandaei*
CN17A0110	*A.caviae*	*A.caviae*	*A.caviae*	*A.caviae*	*A.caviae*	*A.hydrophila*	*A.caviae*	*A.hydrophila*
CN17A0114	*A.veronii*	*A.veronii*	*A.veronii*	*A.sobria*	*A.veronii*	*A.jandaei*	*A.veronii*	*A.jandaei*
CN17A0115	*A.jandaei*	*A.jandaei*	*A.jandaei*	*A.sobria*	*A.veronii*	*A.caviae*	*A.jandaei*	*A.caviae*
CN17A0118	*A.caviae*	*A.caviae*	*A.caviae*	*A.caviae*	*A.caviae*	*A.jandaei*	*A.caviae*	*A.jandaei*
CN17A0119	*A.jandaei*	*A.jandaei*	*A.hydrophila*	*A.sobria*	*A.veronii*	*A.veronii*	*A.jandaei*	*A.veronii*
CN17A0120	*A.veronii*	*A.veronii*	*A.veronii*	*A.sobria*	*A.veronii*	*A.veronii*	*A.veronii*	*A.veronii*
CN17A0122	*A.veronii*	*A.veronii*	*A.veronii*	*A.veronii*	*A.veronii*	*A.veronii*	*A.veronii*	*A.veronii*
CN17A0124	*A.dhakensis*	*A.dhakensis*	*A.hydrophila*	*A.caviae*	*A.hydrophila*	*A.aquariorum*	*A.aquariorum*	*A.aquariorum*
CN17A0125	*A.caviae*	*A.caviae*	*A.caviae*	*A.caviae*	*A.caviae*	*A.caviae*	*A.caviae*	*A.caviae*
CN17A0126	*A.caviae*	*A.caviae*	*A.caviae*	*A.caviae*	*unknown*	*A.caviae*	*A.caviae*	*A.caviae*
CN17A0134	*A.hydrophila*	*A.hydrophila*	*A.hydrophila*	*A.hydrophila*	*A.hydrophila*	*A.hydrophila*	*A.hydrophila*	*A.hydrophila*
CN17A0135	*A.hydrophila*	*A.hydrophila*	*A.hydrophila*	*A.hydrophila*	*A.hydrophila*	*A.hydrophila*	*A.hydrophila*	*A.hydrophila*
CN17A0136	*A.hydrophila*	*A.hydrophila*	*A.hydrophila*	*A.hydrophila*	*A.hydrophila*	*A.hydrophila*	*A.hydrophila*	*A.hydrophila*

CN17A0154	*A.veronii*	*A.veronii*	*A.veronii*	*A.hydrophila*	*A.veronii*	*A.veronii*	*A.veronii*	*A.veronii*
CN17A0158	*A.caviae*	*A.caviae*	*A.caviae*	*A.caviae*	*unknown*	*A.caviae*	*A.caviae*	*A.caviae*
CN17A0164	*A.dhakensis*	*A.dhakensis*	*A.hydrophila*	*A.hydrophila*	*A.hydrophila*	*A.aquariorum*	*A.aquariorum*	*A.aquariorum*
CN17A0166	*A.caviae*	*A.caviae*	*A.caviae*	*A.caviae*	*A.caviae*	*A.caviae*	*A.caviae*	*A.caviae*
CN17A0173	*A.caviae*	*A.caviae*	*A.hydrophila*	*A.caviae*	*A.caviae*	*A.caviae*	*A.caviae*	*A.caviae*
CN17A0176	*A.jandaei*	*A.jandaei*	*A.jandaei*	*A.sobria*	*A.veronii*	*A.jandaei*	*A.jandaei*	*A.jandaei*
CN17A0183	*A.caviae*	*A.caviae*	*A.hydrophila*	*A.caviae*	*A.caviae*	*A.caviae*	*A.caviae*	*A.caviae*
CN17A0191	*A.caviae*	*A.caviae*	*A.hydrophila*	*A.caviae*	*A.caviae*	*A.caviae*	*A.caviae*	*A.caviae*
CN17A0194	*A.dhakensis*	*A.dhakensis*	*A.hydrophila*	*A.hydrophila*	*A.hydrophila*	*A.aquariorum*	*A.aquariorum*	*A.aquariorum*

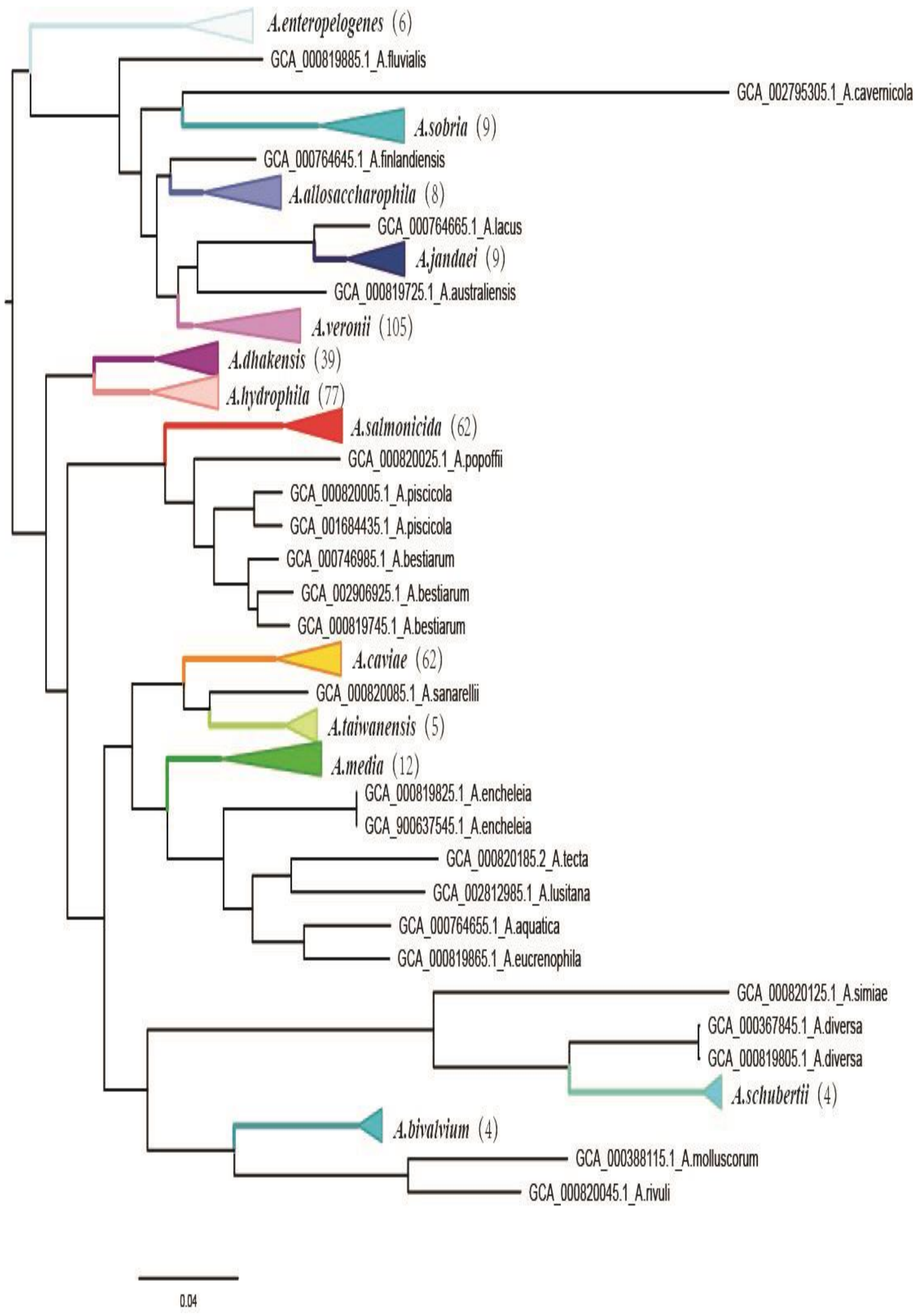

SUPPLEMENTARY FIGURE 1 Maximum likelihood tree results based on genome-wide SNP constructed by 364 strains downloaded from Genbank.
